# Business groups’ internal labour markets and SME labour productivity

**DOI:** 10.1007/s11187-023-00780-4

**Published:** 2023-05-17

**Authors:** Pablo Doucet, Ignacio Requejo, Isabel Suárez-González

**Affiliations:** IME and Business Administration Department, Universidad de Salamanca, Campus Miguel de Unamuno, S/N, 37007 Salamanca, Spain

**Keywords:** Business groups, SMEs, Internal labour markets, Bargaining power, Labour productivity, L22, L25, M51, J21

## Abstract

Labour market regulation constrains small and medium-sized enterprises’ (SMEs) ability to minimize redundant labour. However, it is often neglected that many SMEs might circumvent these constraints by accessing a business group’s internal labour market (ILM). In this study, we analyse whether SMEs with ILM access—i.e., with an increasing number of sister group companies in the same subnational region-industry—enjoy a labour productivity premium and whether this potential productivity premium depends on the bargaining power of labour. Because intra-group reallocation of redundant personnel often involves substantial changes in employment conditions, we argue that the value of ILM access might be reduced when the bargaining power of workers is high as they can oppose the reallocation or demand significant compensation. Using a panel of 119,801 European SMEs during 2011–2019 (639,675 firm-year observations), we find that SMEs with ILM access show relatively higher labour productivity. Further, our findings suggest that this productivity premium is higher in those contexts associated with lower labour bargaining power.

**Supplementary Information:**

The online version contains supplementary material available at 10.1007/s11187-023-00780-4.

## Abstract (plain language summary)

Intra-group reallocation of unused labour can enhance SME labour productivity, but its feasibility and value-adding potential depend on labour bargaining power. This article explores differences in SME labour productivity attributed to internal labour market access, that is, the flexibility to reallocate unproductive labour to other sister companies in the group network. We also analyse if any potential productivity premium associated with internal labour market access depends on the bargaining power of labour. Our statistical analysis shows a labour productivity premium associated with internal labour market access. Furthermore, it is especially significant in contexts associated with low employee bargaining power. For instance, when collective bargaining coverage is low (very low) we predict a labour productivity premium of 3.3% (4.5%) for all SMEs embedded in a network of 10 companies in the same region-industry. Thus, our study contributes to sharpening our understanding of SME productivity and the value they receive from group affiliation.

**Supplementary Information:**

The online version contains supplementary material available at 10.1007/s11187-023-00780-4.

## Introduction

Dismissal costs cause small and medium-sized enterprises (SMEs) to keep underutilized labour (Caballero et al., 2013; Mallett et al., 2019; Van Landuyt et al., 2017). However, it is largely ignored that many SMEs can circumvent these costs by reallocating excess personnel through a business group’s internal labour market (ILM).

A business group can be defined as a set of legally independent firms linked together via ownership ties (Cainelli & Iacobucci, 2011). The literature on business groups has mostly focused on how business groups can add value to their members by functioning as internal capital markets (e.g., Belenzon et al., 2013; Boutin et al., 2013; Kabbach-de-Castro et al., 2022). Little is known though about the benefits of articulating ILMs. Furthermore, the focus of the few existing studies is large corporations (Faccio & O’Brien, 2021; Jung et al., 2019), thus neglecting that many SMEs may benefit from ILM access. Therefore, it is relevant to investigate the value-added of ILM access for SMEs given that (1) they are particularly constrained in adjusting labour on changing opportunities (Williamson, 2000), and because (2) business groups are also ubiquitous in the small business sector (Iacobucci & Rosa, 2005, 2010; Lechner & Leyronas, 2009).

Moreover, previous research does not consider the role of the bargaining power of labour in the feasibility and value-added of ILM access. Anecdotal evidence suggests that reallocation of redundant workers across firms of the same business group often involves substantial changes in employment conditions: employees might learn new skills, lose power within the organization, and see their employment, effort, and pay levels on less favourable terms (Capron & Guillén, 2009; MacKenzie & McLachlan, 2022). This suggests that employee bargaining power might be key in the value-added potential of ILMs.

The aim of this paper is twofold. First, we analyse whether SMEs with ILM access enjoy a labour productivity premium. Second, we investigate whether any potential productivity premium associated with ILM access depends on employee bargaining power. To empirically test these ideas, we collect a sample of 119,801 SMEs (639,675 firm-year observations) across 208 European regions (NUTS 2) covering 21 European countries with available data. Our research context offers an ideal environment for our objectives for two reasons. First, European countries are characterized by an unrivalled accuracy, reliability, and comparability of cross-country SMEs data. Second, it allows us to observe a high level of heterogeneity in the bargaining power of workers, varying across countries, subnational regions, and industries.

Our findings reveal that firms with ILM access have relative higher labour productivity than firms without ILM access. For example, when the average SME is embedded in a network (same subnational region-industry) composed of 10 (20) group firms, its labour productivity increases by 1.5% (3.5%) compared with SMEs without ILM access. In addition, we find that this productivity premium is higher in contexts associated with less employee bargaining power. Specifically, (1) in countries with more permissive labour laws, lower trade union density and collective bargaining power coverage; (2) in subnational regions with lower labour market tightness; and (3) in industries with lower job vacancy rates. For example, when regional labour market tightness is at the mean - 1 S.D. of its distribution—which is associated with lower employee bargaining power—, SMEs embedded in a network composed of 10 (20) group firms see their productivity increase by 3.5% (7%) (again, compared to SMEs without ILM access).

These findings contribute to the literatures on small business and business groups. In the small business literature, we document that ILM access is key to understand labour productivity at the firm level. Few studies consider that many SMEs are embedded in inter-firm networks, which allow them to reduce unproductive labour without using the external labour market. Therefore, future research that investigates SME productivity cannot neglect SME affiliation to these business networks at the industrial-regional level, namely, ILM access. With respect to the business group literature, we provide two main contributions that help to advance our knowledge about the value-added potential of group membership. First, our results suggest that group affiliation per se does not provide the advantages associated with ILM access. In contrast to other affiliation benefits that may apply to all companies in the group (Carney et al., 2011; Holmes et al., 2018; Locorotondo et al., 2012), our findings indicate the need of a certain concentration of companies in the same geographic-industrial area for SMEs to reap the benefits of ILMs. Second, we advance our understanding of how the value-added of being affiliated to these interfirm networks is moderated by the environment. Specifically, we show that contexts in which the bargaining power of workers is higher, either institutionally or through scarcity, erode the ability of ILMs to redeploy unproductive labor and, thus, its associated productivity premium.

This article is organized as follows. Section 2 reviews the literature on business groups’ ILMs and formulates our hypotheses. Section 3 describes our sample and econometric methodology. Sections 4 and 5 present our main results and robustness checks, respectively. Section 6 discusses the empirical evidence and concludes.

## Theory and hypotheses

Whether business groups create or destroy value for group-affiliated firms has been a central topic in the strategic management and industrial organization literature (e.g., Cainelli et al., 2022; Carney et al., 2011; Chu, 2004). One dominant explanation of their value-added potential is that business groups can circumvent market imperfections by establishing internal markets through which affiliates share resources with other sister companies in the group (Khanna & Palepu, 1997, 2000; Leff, 1978). Internal markets in business groups can be especially beneficial to SMEs because they are more resource-constrained than larger firms (Bongini et al., 2021; Motta, 2020; Owalla et al., 2022). For example, the extra resources provided by groups can help SMEs to innovate (Belenzon & Berkovitz, 2010; Guzzini & Iacobucci, 2014), increase exports (Eduardsen et al., 2022; Tajeddin & Carney, 2019), attract financial resources (Cainelli et al., 2020; Iacobucci & Rosa, 2010; Lechner & Leyronas, 2009) and grow (Bamiatzi et al., 2014; Iacobucci, 2002; Iacobucci & Rosa, 2005).

Specifically, ILM access—i.e., being embedded in a network of sister companies that can reallocate labour due to their geographic and industrial proximity—endows SMEs with extra flexibility by offering them an additional avenue to reduce the pool of unproductive labour (Belenzon et al., 2019a; Faccio & O’Brien, 2021; Huneeus et al., 2021). While SMEs without ILM access can only adjust their workforce by hiring and firing workers in the external labour market, SMEs with ILM access can reallocate labour to other sister companies in the group network. This second option induces more flexibility to the extent that employment regulation applies only to labour readjustments that take place through external labour markets, not the ones inside business groups (Belenzon & Tsolmon, 2016; Cestone et al., 2016). Specifically, the EU Directive 96/71/EC^1^ allows affiliated companies to transfer employees without having to bear the costs of dismissal, such as meeting procedural requirements or taking specific actions when issuing the dismissal to the worker. This includes the length of the notice period, the amount of severance pay, and monetary compensation to the worker following an unfair dismissal, as well as the possibility of reinstatement after an unfair dismissal (OECD, 2020).

Consider for example two companies “A” and “B”. On the one hand, A is a company affiliated with a business group and with access to ILM, that is, with the possibility of reallocating workers to other companies in the same region-industry within the group if these workers are not being used in the most productive way in company A. On the other hand, B is a firm that does not enjoy the flexibility derived from ILM access. Now imagine that both companies receive an equivalent negative demand shock. This decrease in firm sales generates a situation of overstaffing, where both companies need to reduce their workforce to realign their labour cost structure with their revenues. Facing this sales decline, firm A enjoys more flexibility to reduce the excess labour generated (and therefore increase labour productivity or a productivity premium) if it can redeploy these workers towards other sister companies through ILM, where they can be fully utilized. The more companies that make up this ILM, the more opportunities firm A will have to reduce unproductive labour without incurring dismissal costs and reputational penalties linked to layoffs (Flanagan & O’Shaughnessy, 2005; Love & Kraatz, 2009; Zyglidopoulos, 2005). In contrast, it is more likely that company B, which does not have the flexibility granted by access to ILM, will retain (some) unproductive workers for longer because the only way it has to trim its workforce is to bear the full costs of layoffs (Autor et al., 2007; Bassanini et al., 2009; Caballero et al., 2013), which include both layoff-related adverse organizational and human effects as well as dismissal costs (e.g., severance payments). If firm A benefits from an optimal ILM allocation of labour across group affiliates when facing changing economic conditions (Cestone et al., 2016), we expect that firm B should exhibit more slack human resources and thus less labour productivity, ceteris paribus.

Redeploying employees from units where they are no longer needed to units where they are needed may also boost productivity by facilitating knowledge spillovers (Chang & Hong, 2000; Lee et al., 2016). When employees move between different companies within a business group, they bring with them the knowledge and expertise they have gained in other sister firms. This can help to transfer knowledge and best practices across the group’s companies. Moreover, this efficiency-based mobility of employees can create opportunities for developing a group-wide shared knowledge base, where employees can gain exposure to different group units and develop a broader understanding of the group’s operations.

Interestingly, intra-group transfers are undertaken in a context of lower information asymmetry concerning workers’ quality. By contrast, external labor markets are fraught with information problems, making it very difficult for hiring firms to predict human capital returns (Chiang & Chiang, 1990; Greenwald, 1986; Jovanovic, 1979). This information problem is, in essence, a version of the classic “market for lemons” phenomenon (Akerlof, 1970). Human capital information asymmetries lead employers to offer lower wages as if they were hiring “lemons” to minimize adverse selection costs. In turn, wage reductions drive high-quality workers (“peaches”) to avoid this job-switching penalty and push them out of the labour market, perpetuating the lemons problem (Chadwick, 2017; Coff, 1997). As a result, firms have a harder time searching for “peaches” in the external labour market, which imposes additional hiring (search/training) costs. In this context, ILM access may enhance labour productivity by giving the option of selecting potential employees (from whom skills and abilities are known) that will better match the resources of the firm (Cestone et al., 2016; Faccio & O’Brien, 2021).

Thus, our baseline hypothesis is that SMEs with the true option to reallocate unproductive labour to other companies in the group should exhibit higher labour productivity than firms without this flexibility:

- H_1_: SMEs with ILM access enjoy a labour productivity premium.

However, the previous arguments to some extent ignore the inherent “stickiness” of human resources (Mishina et al., 2004; Penrose, 1959; Voss et al., 2008). Research results indicate that unused labour is difficult to redeploy, especially when employees’ bargaining power constrains managers’ decision-making and discretion. In terms of employment relationships, bargaining power is the capacity of a party to produce an agreement on its own terms (Dencker, 2009; Pfeffer & Salancik, 1978; Phillips & Sørensen, 2003). The relative power of employees varies with labour market institutions (as manifested in employment laws and collective bargaining agreements) and labour market conditions (tightness/slackness) (Dencker, 2009; Hansen, 1970; Vanacker et al., 2017). For example, in a tight labour market—i.e., when unemployment is low—, employees have greater bargaining power as they face lower mobility costs outside organizations (search, bargaining, and switching costs) (Campbell et al., 2012; Hansen, 1970). It therefore follows that business groups will face more difficulties to conduct productivity-enhancing reallocations when labour market conditions are tight. Through labour legislation, the bargaining power of workers is determined by the procedural requirements that the firm must follow before or when dismissing the worker, regulations regarding notice and severance pay, the framework for defining unfair dismissal, and the enforcement of unfair dismissal regulations (OECD, 2020). For example, employees have greater bargaining power when the amount of severance pay is increased, the possibility of reinstatement following unfair dismissal is higher, and when they are released from the burden of proof when filing a complaint for unfair dismissal.

Scholars have used relative bargaining power theory to advance our understanding on how differences in the distribution of power among key stakeholders influence restructuring decisions. For instance, Van Essen et al. (2013) find that stronger employee rights restrict the ability of blockholders to pursue value-enhancing strategies such as reducing the pool of redundant employees. Capron and Guillén (2009) find that stricter employment protection laws (EPL) regimes significantly constrain an acquirers’ ability to redeploy employees from the target. Another example is Vanacker et al. (2017), who find that stronger EPL restrict managers’ discretion to reduce underutilized human resources.

In our context, power differentials determine if (and at what cost) business groups can reallocate redundant workers across group-affiliated firms. Intra-group reallocations of redundant personnel denote a clear shift in the employment relationship, as firms exercise their power not only to break the current employment relationship but also to change its terms considerably. When redundant personnel are transferred across group affiliates, they must often learn new skills, may lose power within the organization, and see their employment, effort and pay levels on less favourable terms (Capron & Guillén, 2009; MacKenzie & McLachlan, 2022). Consequently, if employee bargaining power is high, target employees are likely to oppose internal reallocation or demand a more aggressive compensation for their mobility—which limit ILMs’ ability to minimize unproductive labour.

In sum, if the productivity premium associated with ILM access depends on group’s discretion to reshape their pool of unproductive labour via intra-group reallocations, we expect to see this productivity premium attenuated when employees have strong bargaining power vis-à-vis the business group. Thus, we propose that:

- H_2_: The productivity premium associated with ILM access is higher/lower when employee bargaining power is lower/higher.

## Data and methods

We build our sample using Amadeus, a database developed by Bureau van Dijk. This database provides detailed information on ownership links as well as standardized financial information of European firms. We apply several filters to arrive at our final sample. First, following other studies on SMEs and business groups (e.g., Guzzini & Iacobucci, 2014) and EU’s definition of SMEs,^2^ we restrict our sample to firms employing less than 250 employees.^3^ Second, SMEs from the financial sector are excluded as the returns in this sector are not comparable to returns in the other sectors of the economy. Finally, following Desai et al. (2003), we remove firms with fewer than 20 employees, a cut-off point that ensures comparable samples across countries and avoids radical changes in labour productivity due to employee turnover (De Meulenaere et al., 2021).^4^ Based on these criteria, we have an unbalanced panel of 119,801 European SMEs during 2011–2019 (639,675 firm-year observations).

### Dependent variable

In accordance with previous studies on the value-added function of ILMs (e.g., see Belenzon & Tsolmon, 2016; Tate & Yang, 2015), our dependent variable is labour productivity, measured as the natural logarithm of total sales divided by the number of employees.

### Key explanatory variables

#### ILM access

Group ILM access is a count variable of the number of sister companies with which the focal firm shares industry-subnational region. It measures the extent to which an affiliate can redeploy workers from (to) other group members, and it is calculated in three steps. First, we identify all the ownership links between different European firms (control is achieved by at least 50% ownership).^5^ Then, a name-matching procedure identifies all legally distinct firms controlled by the same ultimate owner (Belenzon & Berkovitz, 2010; Faccio & O’Brien, 2021). Finally, having established all ownership links, we calculate the number of same-industry group-affiliated firms (two-digit SIC code) in each region (NUTS 2).^6^ Firms that fulfil these conditions, unlike standalone and other group-affiliated firms, do not face the (undoubtedly too high) adjustment costs of moving workers outside their home region and/or outside their field of expertise.

#### Employee bargaining power

We employ five different measures of labour bargaining power, which have already been used in previous research (e.g., Capron & Guillén, 2009; Van Landuyt et al., 2017; Vanacker et al., 2017).

##### EPL index

Employee bargaining power is established in national laws. Our measure of country EPL is the Organisation for Economic Cooperation and Development (OECD) employment dismissal protection index. This index measures the difficulty of dismissing workers based on the following dimensions: difficulty of dismissal, notice and severance pay requirements, procedural inconveniences of dismissal, and additional provisions for collective dismissals. The measure ranges from 0 to 6, with higher values indicating higher power of labour.

##### Collective bargaining coverage

The OECD adjusted collective bargaining coverage rate is defined as the number of employees covered by a collective agreement in force as a proportion of the number of eligible employees (i.e., the total number of employees minus the number of employees legally excluded from the right to bargain). The higher this proportion, the higher the bargaining power of employees.

##### Trade union density

The trade union density, also provided by the OECD, is defined as the number of net union members (i.e., excluding those who are not in the labour force, unemployed, and self-employed) as a proportion of the number of employees. Higher trade union density translates into higher employee’s bargaining power.

##### Job vacancy rate

The annual job vacancy rate (JVR) published by Eurostat. It measures the proportion (expressed as a percentage) of total posts that are vacant in each country-industry-year (based on the NACE Rev. 2 codes for the industry classification). A job vacancy is defined as a paid post (newly created, unoccupied, or about to become vacant) for which the employer is taking active steps to find a suitable candidate from outside the enterprise (even being prepared to take more steps if needed) and which it intends to fill either immediately or in the near future. A higher JVR is associated with less mobility costs as it is less costly for workers to search for and switch to alternative jobs and they have more power to negotiate with their current/potential employers.

##### Regional labour market tightness

Labour market conditions—which are said to be either slack or tight—affect employee’s bargaining position vis-à-vis employer interests. The term ‘slack’ describes the unmet demand for paid labour within a given population. When the labour market is slack, workers remain involuntarily unemployed or alternatively work fewer hours than they wish. This generally results in a situation in which employee bargaining power in terms of wages and employment conditions is weaker. Labour market ‘tightness’ describes the exact opposite situation. We measure labour market tightness as the level of labour market slack multiplied by -1. Data on the region-level labour market slack (in which the unemployment rate is a major component) are gathered from the Eurostat database.^7^

In all five cases, we use mean-centred variables to reduce multicollinearity concerns when interacting this variable with ILM access.

### Control variables

Building on previous studies that have used labour productivity as an outcome variable (e.g., Belenzon & Tsolmon, 2016; Motta, 2020; Tate & Yang, 2015) as well as on our specific research context, we include a set of control variables in the regression analyses to mitigate the risk of confounding effects: firm’s size, calculated as the natural log of the firm’s employees; firm’s age, measured as the number of years since the firm was established; firm’s capital intensity, defined as the ratio of tangible fixed assets over the number of full-time-equivalent employees; sales growth, calculated by dividing the current year’s revenues by the prior year’s revenues and subtracting 1; the number of group firms outside the region-industry, computed by subtracting ILM access from the total number of affiliates in a given business group; firm leverage, calculated as the ratio of debt to equity; industry labour productivity, measured as the ratio of turnover per person employed in each country-industry-year, retrieved from Eurostat’s Structural Business Statistics (SBS)^8^; the level of economic development of the region, measured as the natural logarithm of regional GDP per capita; the level of competitiveness of the region, using the European Regional Competitiveness Index (RCI) for the 2011–2019 period^9^; and country, industry (two-digit SIC codes), and year fixed effects. All measures are lagged one year to mitigate potential simultaneity concerns.

### Descriptive statistics

Table 1 presents the means, standard deviations, and main percentiles of the variables. Interestingly, 17% of the firm-year observations in the sample correspond to firms with at least one sister company operating in the same region-industry. The correlation matrix of the variables used in the regression analyses is provided in Table A1 (Appendix).

**Table 1:** Summary statistics of main variables

	Mean	S.D.	P10	P50	P90
Labour productivity	5.06	0.98	3.80	5.06	6.30
ILM access	0.46	1.78	0.00	0.00	1.00
EPL index	-0.02	0.32	-0.43	-0.01	0.44
Collective bargaining coverage	0.02	0.22	-0.43	0.08	0.20
Trade union density	-0.01	0.16	-0.16	0.04	0.24
Job vacancy rate	-0.04	1.00	-0.81	-0.31	1.09
Labour market tightness	-0.01	0.09	-0.15	0.01	0.09
Firm size	4.04	0.65	3.30	3.89	5.01
Firm age	3.04	0.74	2.08	3.14	3.83
Sales growth	0.07	0.34	-0.13	0.03	0.26
Capital intensity	4.78	1.13	3.30	4.84	6.14
Leverage	3.69	1.87	1.23	4.02	5.72
Num. group firms outside the region-industry	2.08	5.80	0.00	0.00	6.00
Industry productivity	5.10	0.80	4.04	5.08	6.12
Regional GDP	11.28	0.98	10.05	11.18	12.81
Regional competitiveness index	-0.14	0.50	-0.79	-0.18	0.59

### Econometric specification

Given the panel data structure of the sample, fixed effects or random effects models could be used to account for non-independent error terms (Greene, 2011). We use a random effects specification because our key independent variables are time invariant and could not be accommodated in a fixed effects specification.^10^ In contrast, a random effects specification allows us to estimate the impact of time invariant variables. Thus, our preferred estimation method is generalized least squares (GLS) random effects (the “xtreg” command in Stata). Standard errors are adjusted using the Huber/White/sandwich estimator to provide reliable estimates in the presence of heteroskedasticity and autocorrelation (Wooldridge, 2002).

## Results

Table 2 contains the regressions that allow us to test H1 and H2. Model 1 includes only control variables. In Model 2, in addition to our controls, we add ILM access as an independent variable to test H1. In Models 3–7, we include two-way interaction terms between ILM access and the variables representing employee bargaining power to test H2.

**Table 2:** Main results

DV:	Labour productivity						
Employee bargaining power measure:			EPL strictness	Collective bargaining coverage	Trade union density	Job vacancy rate	Regional labour market tightness
Model:	(1)	(2)	(3)	(4)	(5)	(6)	(7)
Firm size	0.059^***^	0.059^***^	0.060^***^	0.066^***^	0.059^***^	0.061^***^	0.058^***^
	(0.003)	(0.003)	(0.003)	(0.003)	(0.003)	(0.003)	(0.003)
Firm age	0.051^***^	0.051^***^	0.050^***^	0.047^***^	0.052^***^	0.051^***^	0.050^***^
	(0.002)	(0.002)	(0.002)	(0.002)	(0.002)	(0.002)	(0.002)
Sales growth	0.215^***^	0.215^***^	0.213^***^	0.209^***^	0.208^***^	0.216^***^	0.215^***^
	(0.003)	(0.003)	(0.003)	(0.003)	(0.003)	(0.003)	(0.003)
Capital intensity	0.372^***^	0.372^***^	0.375^***^	0.387^***^	0.381^***^	0.373^***^	0.372^***^
	(0.002)	(0.002)	(0.002)	(0.002)	(0.002)	(0.002)	(0.002)
Num. group firms outside the region-industry	0.008^***^	0.006^***^	0.006^***^	0.006^***^	0.007^***^	0.007^***^	0.006^***^
	(0.000)	(0.000)	(0.000)	(0.000)	(0.000)	(0.000)	(0.000)
Leverage	-0.005^***^	-0.005^***^	-0.005^***^	-0.006^***^	-0.005^***^	-0.006^***^	-0.005^***^
	(0.000)	(0.000)	(0.000)	(0.001)	(0.001)	(0.000)	(0.000)
Industry labour productivity	0.243^***^	0.243^***^	0.240^***^	0.248^***^	0.231^***^	0.244^***^	0.244^***^
	(0.005)	(0.005)	(0.006)	(0.006)	(0.006)	(0.006)	(0.005)
Regional GDP	0.015^***^	0.014^***^	0.014^***^	0.012^***^	0.022^***^	0.020^***^	0.011^***^
	(0.002)	(0.002)	(0.002)	(0.002)	(0.003)	(0.002)	(0.002)
Regional competitiveness index	0.132^***^	0.132^***^	0.131^***^	0.146^***^	0.136^***^	0.100^***^	0.092^***^
	(0.005)	(0.005)	(0.005)	(0.005)	(0.005)	(0.005)	(0.006)
**ILM access**		0.009^***^	0.009^***^	0.011^***^	0.012^***^	0.009^***^	0.009^***^
		**(0.001)**	**(0.001)**	**(0.001)**	**(0.001)**	**(0.001)**	**(0.001)**
Bargaining power			0.031^***^			0.018^***^	0.692^***^
			(0.003)			(0.001)	(0.034)
**ILM access × Bargaining power**			-0.006^***^	-0.024^***^	-0.040^***^	-0.003^***^	-0.101^***^
			**(0.002)**	**(0.004)**	**(0.006)**	**(0.001)**	**(0.011)**
Constant	1.711^***^	1.717^***^	1.702^***^	1.783^***^	1.787^***^	1.665^***^	1.726^***^
	(0.255)	(0.255)	(0.254)	(0.242)	(0.258)	(0.257)	(0.257)
Observations Firms	639,675	639,675	625,919	532,269	527,996	609,962	639,548
	119,801	119,801	119,313	109,694	101,853	113,150	119,776
Wald chi square	287,346.276	287,787.696	284,657.374	263,498.899	244,185.147	277,232.692	291,514.908
p-value	0.000	0.000	0.000	0.000	0.000	0.000	0.000
R^2^	0.719	0.719	0.718	0.724	0.713	0.722	0.720

This table presents the results on the relationship between ILM access and labour productivity, and how it varies with employee bargaining power. We ran GLS random effects panel data models in which standard errors (in parentheses) are robust to heteroscedasticity and allow for serial correlation through clustering by firms. All models include country, industry, and year fixed effects. The direct effects of Collective bargaining power (in Model 4) and Trade union density (in Model 5) are omitted due to collinearity problems with the country fixed effects. Bold font highlights variables or interactions of interest

The results for the control variables provide some interesting insights. Firm size is positively related with labour productivity (*b* = 0.059, *p* < 0.001). An increase from the mean - 1 S.D. to the mean + 1 S.D. in firm size increases labour productivity by about 1.6% for the average firm. Firm age is also positively related with labour productivity (*b* = 0.051, *p* < 0.001). An increase from the mean - 1 S.D. to the mean + 1 S.D. in firm age increases labour productivity by about 1.8% for the average firm. Sales growth is positively related with labour productivity (*b* = 0.215, *p* < 0.001). An increase from the mean - 1 S.D. to the mean + 1 S.D. in sales growth increases labour productivity by about 3.22% for the average firm. Capital intensity is positively related and has an important economic effect on labour productivity (*b* = 0.372, *p* < 0.001). An increase from the mean - 1 S.D. to the mean + 1 S.D. in capital intensity increases labour productivity by about 20.4% for the average firm. An increasing number of sister companies outside the region-industry is positively related with labour productivity (*b* = 0.008, *p* < 0.001). If this variable increases from the mean - 1 S.D. to the mean + 1 S.D., labour productivity increases by about 2% for the average firm. Firm leverage is negatively related with labour productivity (*b* = -0.005, *p* < 0.001). An increase from the mean - 1 S.D. to the mean + 1 S.D. in firm leverage decreases labour productivity by about -0.4% for the average firm.

With respect to the industry-level controls, industry labour productivity is positively related with labour productivity (*b* = 0.243, *p* < 0.001). An increase from the mean - 1 S.D. to the mean + 1 S.D. in industry labour productivity increases labour productivity by about 8.3% for the average firm. Regarding the regional-level controls, regional GDP is positively related with labour productivity (*b* = 0.015, *p* < 0.001). An increase from the mean - 1 S.D. to the mean + 1 S.D. in regional GDP increases labour productivity by about 0.4% for the average firm. The regional competitiveness index is also positively related with labour productivity (*b* = 0.132, *p* < 0.001). An increase from the mean - 1 S.D. to the mean + 1 S.D. in this index increases labour productivity by about 2.6% for the average firm.

As shown in Model 2 (Table 2), ILM access is positively related and has an economically significant impact on labour productivity (*b* = 0.009, *p* < 0.001). When all other variables are held at their means, our model predicts that SMEs embedded in a network (same subnational region-industry) composed of 10 (20) group firms, each of them sees their labour productivity increase by 1.5% (3.5%) compared with SMEs without ILM access. These findings support H1, which proposes that ILM access is associated with a labour productivity premium.

In Models 3–7 in Table 2, we test H2, which suggests that the labour productivity expected in H1 is higher when employee bargaining power is lower. In Models 3–7, we find that the coefficients of the interactions between ILM access and our proxies for labour bargaining power are negative and statistically significant. These findings suggest that the productivity premium associated with ILM access is higher (1) in countries with less stringent labour laws, lower trade union density and collective bargaining power coverage; (2) in subnational regions with lower labour market tightness; and (3) in industries with lower job vacancy rates. For example, as shown in Model 3 of Table 2, the coefficient of ILM access × EPL strictness (*b* = -0.006, *p* < 0.001) means that, when EPL strictness is at the mean + 1 S.D. of its distribution, the average SME embedded in a network of 10 (20) group firms sees its productivity increase by 1.4% (2.8%) with respect to its non-ILM access counterpart. In contrast, when EPL strictness is at the mean - 1 S.D. of its distribution, the average SME embedded in a network of 10 (20) group firms experiences a productivity increase of 2.2% (4.2%) with respect to its non-ILM access counterpart. Put differently, the lower the EPL strictness, the higher the productivity premium granted by ILM access.

**Table 3:** Percentage increase in labour productivity of the average SME with ILM access with respect to its non-ILM access counterpart, by level of employee bargaining power

Employee bargaining power measure	Distribution point	ILM access = 10	ILM access = 20
EPL strictness	µ - 2σ	**2.4**	**4.8**
	µ - σ	**2.2**	**4.2**
	µ + σ	1.4	2.8
	µ + 2σ	1	2
Collective bargaining coverage	µ - 2σ	**4.5**	**8.9**
	µ - σ	**3.3**	**6.5**
	µ + σ	1	1.8
	µ + 2σ	-0.2	-0.6
Trade union density	µ - 2σ	**5.1**	**10.4**
	µ - σ	**3.7**	**7.6**
	µ + σ	1	2.1
	µ + 2σ	-0.2	-2.1
Job vacancy rate	µ - 2σ	**2.8**	**5.8**
	µ - σ	**2.2**	**4.6**
	µ + σ	1.2	2.2
	µ + 2σ	0.6	1.4
Regional labour market tightness	µ - 2σ	**6.1**	**12.2**
	µ - σ	**3.4**	**6.8**
	µ + σ	0	0.2
	µ + 2σ	-1.5	-3.3

Bold font highlights linear predictions that correspond with contexts associated with low employee bargaining power

Overall, results displayed in Table 3 confirm that the moderating effect of employee bargaining power on the productivity premium associated with ILM access is not only statistically significant but also economically meaningful.

## Additional analyses and sensitivity checks

### Additional evidence regarding the ILM mechanism

Our H1 suggests that SMEs with ILM access enjoy a productivity premium because they can redeploy unused labour to other sister companies where they can be fully utilized. According to this theoretical mechanism, the productivity premium associated with ILM access should be more evident when comparing SMEs with ILM access that face declining revenues with their non-ILM access counterparts. One immediate consequence of revenue decline is the creation of personnel redundancies. However, revenue decline should be more likely to result in unused labour in SMEs without ILM access, as they do not have the ability to conduct efficiency-based intra-group reallocations. We test these arguments, which suggest causality, in Table 4, where we interact ILM access with negative revenue shocks in t, t-1, and t-2. As shown, the pattern of results reveals that the productivity premium is more evident when SMEs are facing negative demand shocks, supporting the superior ability of SMEs with ILM access to reduce unused labour.

**Table 4:** Evidence regarding the ILM mechanism

DV:	Labour productivity		
Model:	(1)	(2)	(3)
Firm size	0.040^***^	0.050^***^	0.057^***^
	(0.002)	(0.003)	(0.003)
Firm age	0.001	0.004	0.006^*^
	(0.002)	(0.002)	(0.003)
Capital intensity	0.364^***^	0.363^***^	0.362^***^
	(0.002)	(0.002)	(0.003)
Num. group firms outside the region-industry	0.007^***^	0.007^***^	0.007^***^
	(0.000)	(0.000)	(0.000)
Leverage	-0.006^***^	-0.007^***^	-0.007^***^
	(0.000)	(0.000)	(0.000)
Industry labour productivity	0.242^***^	0.226^***^	0.222^***^
	(0.005)	(0.006)	(0.006)
Regional GDP	0.014^***^	0.010^***^	0.012^***^
	(0.002)	(0.002)	(0.003)
Regional competitiveness index	0.139^***^	0.158^***^	0.155^***^
	(0.005)	(0.005)	(0.006)
**ILM access**	0.008^***^	0.007^***^	0.007^***^
	**(0.001)**	**(0.001)**	**(0.001)**
**Revenue shock**	-0.122^***^	-0.127^***^	-0.134^***^
	**(0.001)**	**(0.001)**	**(0.001)**
**ILM access × Revenue shock**	0.001^***^	0.002^***^	0.002^***^
	**(0.000)**	**(0.000)**	**(0.000)**
**Revenue shock**_**t-1**_		-0.063^***^	-0.069^***^
		**(0.001)**	**(0.001)**
**ILM access × Revenue shock**_**t-1**_		0.001^***^	0.002^***^
		**(0.000)**	**(0.000)**
**Revenue shock**_**t-2**_			-0.046^***^
			**(0.001)**
**ILM access × Revenue shock**_**t-2**_			0.001^*^
			**(0.000)**
Constant	2.095^***^	2.344^***^	2.394^***^
	(0.239)	(0.241)	(0.226)
Observations	639,675	553,373	471,867
Firms	119,801	111,227	102,341
Wald chi square	320,695.130	299,241.172	273,525.802
p-value	0.000	0.000	0.000
R^2^	0.723	0.725	0.726

Revenue shock, Revenue shock_t-1_, and Revenue shock_t-2_ are operationalized as dummy variables that take the value of 1 if there is a reduction (and 0 in the absence of a sales reduction) in sales from t-1 to t, from t-2 to t-1, and from t-3 to t-2, respectively. We ran GLS random-effects panel models in which standard errors (in parentheses) are robust to heteroscedasticity and allow for serial correlation through clustering by firms. All models include country, industry, and year fixed effects. Bold font highlights variables or interactions of interest

### Ruling out intra-group knowledge spillover explanations

This paper documents that SMEs with ILM access enjoy a labour productivity premium, particularly in contexts associated with lower labour bargaining power. However, it is possible that this premium can be explained by the functioning of the business group as an effective organization in expanding the knowledge resources of a firm. Specifically, beyond the knowledge spillovers associated with the ILM mechanism described in this article, labour productivity can be enhanced through intra-group knowledge spillovers resulting from international ties that benefit from foreign technical advances (Coe & Helpman, 1995) and transfers of new technology or innovation among group members (Belenzon & Berkovitz, 2010).

We believe that our results are not explained by these intra-group knowledge spillovers for at least two reasons. Firstly, our analysis in Table 4, where we examine the ILM mechanism, reveals that when sales decline, SMEs without ILM access are more likely to experience a decrease in labour productivity due to excess personnel. Secondly, our control variable ‘Num. group firms outside the region-industry’ already controls for the positive benefits that group-affiliated firms receive from the knowledge pool of the mother group.

Nonetheless, we have conducted a series of tests to rule out the possible effects of intra-group knowledge spillovers related to international ties and transfers of technology and innovation among group members. On the one hand, we have explored whether the productivity premium associated with ILM access diverges between knowledge-intensive activities (KIA) and non-KIA.^11^ Inter-firm knowledge spillovers are expected to be higher in KIA because these activities involve the creation, dissemination, and use of knowledge as their core inputs. In such activities, group-affiliated firms are likely to interact more frequently with other sister companies, which can lead to increased opportunities for knowledge exchange and collaboration. As shown in Model 2 of Table 5, results are inconsistent with the knowledge spillover hypothesis: we find that the ILM access productivity premium is higher in non-KIA. The coefficient of ILM access × KIA (*b* = -0.012, *p* < 0.001) means that, for non-KIA, the average SME embedded in a network of 10 (20) group firms sees its productivity increase by 2% (4%) with respect to its non-ILM access counterpart. In contrast, for KIA, the average SME embedded in a network of 10 (20) group firms experiences a productivity change of 0% (-0.2%) with respect to its non-ILM access counterpart. In other words, the productivity premium associated with ILM access only occurs in non-KIA sectors. These results are consistent with our findings, suggesting a labour productivity premium associated with ILM access when labour bargaining power is low. In general, we would expect to see more bargaining power for labour in KIA than in non-KIA. This is because KIA typically require highly skilled and specialized workers with unique knowledge and expertise that is valuable to firms. In these sectors, workers with specialized skills and knowledge are in relatively short supply, which can give them more bargaining power in situations where they must be reallocated within a group and their labour conditions modified, limiting ILM’s ability to conduct efficiency-based intra-group reallocations. In contrast, in non-KIA sectors, workers are typically more interchangeable and can be more easily replaced (Blatter et al., 2012), which can weaken labour bargaining power and thus enhance the group’s ability to activate ILMs.

**Table 5:** Ruling out intra-group knowledge spillover explanations

DV:	Labour productivity					
Model:	(1)		(2)		(3)	
	b	se	b	se	b	se
Firm size	0.059^***^	(0.003)	0.059^***^	(0.003)	0.058^***^	(0.003)
Firm age	0.051^***^	(0.002)	0.051^***^	(0.002)	0.051^***^	(0.002)
Sales growth	0.215^***^	(0.003)	0.215^***^	(0.003)	0.215^***^	(0.003)
Capital intensity	0.372^***^	(0.002)	0.372^***^	(0.002)	0.372^***^	(0.002)
Leverage	-0.005^***^	(0.000)	-0.005^***^	(0.000)	-0.005^***^	(0.000)
Num. group firms outside the region-industry	0.008^***^	(0.000)	0.007^***^	(0.000)		
Industry labour productivity	0.243^***^	(0.005)	0.242^***^	(0.005)	0.244^***^	(0.005)
Regional GDP	0.015^***^	(0.002)	0.014^***^	(0.002)	0.013^***^	(0.002)
Regional competitiveness index	0.132^***^	(0.005)	0.132^***^	(0.005)	0.132^***^	(0.005)
**KIA**	**0.429**	**(0.255)**	**0.433**	**(0.255)**		
**ILM access**			0.011^***^	**(0.001)**	0.009^***^	**(0.001)**
**ILM access × KIA**			-0.012^***^	**(0.002)**		
**KIA group ties**					0.003^*^	**(0.001)**
**International group ties**					0.016^***^	**(0.001)**
Constant	1.711^***^	(0.255)	1.716^***^	(0.255)	1.720^***^	(0.254)
Observations	639,678		639,678		639,678	
Firms	119,802		119,802		119,802	
Wald chi square	287,346.477		287,897.357		288,639.161	
p-value	0.000		0.000		0.000	
R^2^	0.719		0.720		0.720	

The acronym KIA refers to knowledge-intensive activities. An activity is classified as knowledge intensive if more than 33% of the total employment in that activity is comprised of tertiary-educated individuals. Using the NACE Rev. 2 classification at the 2-digit level, the full list of KIA sectors includes: 09, 19, 21, 26, 51, 58, 59, 60, 61, 62, 63, 64, 65, 66, 69, 70, 71, 72, 73, 74, 75, 78, 79, 84, 85, 86, 90, 91, 94, and 99. We estimated GLS random-effects panel models in which standard errors (in parentheses) are robust to heteroscedasticity and allow for serial correlation through clustering by firms. All models include country, industry, and year fixed effects. Variables or interactions of interest are highlighted in bold font

Finally, in Model 3 of Table 5, we have replaced the variable ‘Num. group firms outside the region-industry’ with more fined-grained measures of intra-group knowledge spillovers. Specifically, we have added the ‘KIA group ties’ and ‘International group ties’ variables to control for knowledge spillovers stemming from being tied to a business group with increasing activity in KIA and increasing foreign operations.^12^ As shown, the coefficient for ILM access remains positive, statistically significant, and economically significant even after including these additional more fine-grained measures of intra-group knowledge spillovers, which have also been found to have a positive impact on labour productivity in previous research (Cainelli et al., 2022; Lee et al., 2016).

Overall, both tests suggest that intra-group knowledge spillovers coming from international ties and innovation transfers are unlikely to be the main driver of the labour productivity premium that we have reported for firms with ILM access.

### Alternative measure for ILM access

Models 1 to 7 in Tables A2, A3, and A4 (Appendix) confirm that our findings do not change if we use alternative definitions of ILM access that are based on different thresholds of industry/geographic proximity. Specifically, instead of measuring ILM access as the number of sister group companies in the same NUTS2 and two-digit SIC codes, we use the number of sister group companies in the same NUTS2 and three-digit SIC codes (Table A2), the number of sister group companies in the same NUTS1 and two-digit SIC codes (Table A3), and the number of sister group companies in the same country and two-digit SIC codes (Table A4).

Although we believe that these measures accurately capture the flexibility granted by an ILM, it could be argued that ILM measures at the regional or country level have certain limitations. On the one hand, defining the ILM access variable at the regional level may result in certain distortions if many group-affiliated firms are organized around the border of two or more regions within the same country. On the other hand, computing the ILM access variable at the country level may not accurately capture the flexibility to redeploy workers within the ILM, as some countries are very large in geographical area and thus make worker reallocations unviable. To mitigate this potential distortion in the calculation of ILM access, we examine whether the productivity premium observed in Table 2 persists when employing the ILM access variable constructed at the country-two-digit SIC code level while excluding countries with the largest geographical areas from the sample. Specifically, models 1 and 2 in Table A5 exclude the three largest countries in the sample; models 3 and 4 exclude the five largest countries; and models 5 and 6 exclude the seven largest countries.^13^ In this way, we ensure that both assumptions about the feasibility of intra-group reallocation of workers are met: on the one hand, it is difficult to move workers outside the established ILMs; on the other hand, we ensure a certain spatial concentration that makes staff redeployment within the group feasible.

Finally, in Table A6, we measure ILM access in terms of employees rather than sister firms. Specifically, we calculate ILM access by subtracting the number of employees in the focal firm from the total number of employees in the same industry-subnational region. Overall, all these tests lead to results that are similar to our main findings, which have been presented in Table 2.

### CEM-matched sample

We perform a coarsened exact matching (CEM) analysis that accounts for differences in observable characteristics between SMEs with versus those without ILM access. In this way, we mitigate concerns about unobservable characteristics that may be linked to the observable characteristics. This matching technique allows us to create a matched sample, where each treatment observation (i.e., SMEs that have at least one sister company) is matched with a control observation (i.e., SMEs that do not have any sister company) that have similar observable characteristics. CEM is like propensity score matching but requires fewer post-estimation assumptions (Iacus et al., 2012). We apply this technique using the Stata command *cem*. Specifically, we match firm years based on firm assets, labour costs, and the industry (four-digit SIC code). We have used the “k2k” option to allow CEM to produce a matching result that has the same number of treated and control units within each matched stratum. Our final sample consist of 40,263 matched firms. We then rerun our regressions on the matched sample. As Table A7 reports, the results remain robust to the use of this matched sample.

## Discussion and conclusions

Employment regulation limits SMEs’ ability to reduce unused labour. Consequently, SMEs often retain unproductive workers whose wage exceeds their productivity. These constraints may be circumvented when an SME belongs to a business group and has access to its ILM. In this study, we investigate whether SMEs with ILM access enjoy a labour productivity premium. Moreover, because intragroup reallocation of redundant workers often involves changing employment conditions, we investigate whether the aforementioned potential productivity premium depends on employee bargaining power.

Using a comprehensive dataset that covers 119,801 European SMEs (2011–2019), we find strong support for our baseline hypothesis that SMEs with greater access to a group’s ILM—which occurs when there is a higher concentration of sister companies in the same industry-region—have higher labour productivity. This result suggests that the flexibility provided by ILM access to make efficiency-based labour adjustments that are not subject to EPL regulations enhances SME productivity. This efficient functioning of ILMs may further boost productivity by promoting knowledge spillovers within the group if redeployed employees bring with them valuable knowledge and best practices gained in other sister companies. Further, we find strong evidence that this productivity premium is attenuated by employee bargaining power. These findings are consistent with a growing research stream that highlights the role of business group affiliation in explaining SME behaviour and performance (e.g., Eduardsen et al., 2022; Guzzini & Iacobucci, 2014; Lechner & Leyronas, 2009). We add to this literature by focusing on ILM access as an important element for understanding SME labour productivity. Our results, jointly with the prevalence of group-affiliated SMEs with ILM access (around 17% of our sample have at least one close sister company), underscore the need to consider the embeddedness within these interfirm networks when analyzing SME productivity.

In addition, we contribute to the nascent study of the value-added function of groups’ ILMs (together with Belenzon & Tsolmon, 2016; Cestone et al., 2016, Faccio & O’Brien, 2021; Huneeus et al., 2021; Jung et al., 2019). The novelty of our results is that it is not belonging to a business group per se that matters; instead, it is the increasing option to redeploy employees among other sister companies that is important. In fact, in contrast to previous studies, we identify an asymmetry between group-affiliated SMEs with ILM access and group-affiliated SMEs without ILM access.

Moreover, no previous research has considered the bargaining power of labour in the feasibility and value-added of intragroup reallocations. The focus of previous works has been on how ILM access adds value in the presence of external labour market frictions, namely, hiring and firing costs imposed by EPL (Belenzon & Tsolmon, 2016; Cestone et al., 2016; Faccio & O’Brien, 2021). Because intragroup labour adjustments are exempt from EPL, these studies have observed that stricter EPL put group-affiliated firms in a better position (compared to stand-alone firms) to reduce their pool of unused labour. Thus, it follows from this research that group affiliation increases labour productivity when EPL is stricter. Interestingly, our results show the opposite effect: the labour productivity premium is higher in countries with low EPL strictness. Nevertheless, we believe that this finding is not incompatible with the arguments laid out in previous studies. The flexibility to adjust labour without incurring in EPL penalties is the core mechanism through which ILM access adds value for member firms. However, in addition to high external adjustment costs, a high value in the EPL index is related with a stronger employee bargaining position, which may hinder intragroup transfers of redundant personnel. Therefore, our results suggest that, although avoiding EPL penalties is the core value-adding mechanism of ILM access, too strict EPL can neutralize the feasibility and value-added of this type of organizational market.

Finally, we answer the calls for studying the benefits/costs of group affiliation in developed economies, which have been typically neglected (Aguilera et al., 2020; Carney et al., 2011; Holmes et al., 2018). It is generally thought that the ‘institutional voids’ (IV) perspective (Carney et al., 2018; Leff, 1978)—one of the most widely used theories in the business group literature—is unable to explain (1) why business groups have not faded away or (2) why affiliated firms continue to have a more than acceptable economic performance in advanced economies (Cainelli et al., 2022; Carney et al., 2011; Belenzon & Berkovitz, 2008). This limitation justifies the lack of research on the consequences of group affiliation in such a context (Locorotondo et al., 2012). Here, our study follows recent extensions of the IV perspective that propose a more general transaction costs story of business groups: groups’ internal markets can overcome the failures of arms-length market contracting in any type of economy (see, for instance, Belenzon et al., 2013; Cainelli et al., 2020, 2022). Leveraging on this idea, we challenge the conventional wisdom that business groups are gap-fillers only in less developed settings. In this way, our results reconcile the conflicting predictions of the IV theory with the dominant economic role of business groups in European countries (Cainelli et al., 2011; Carney et al., 2011; Colpan & Hikino, 2018).

### Limitations and further research

Our paper has some limitations that open new avenues for future works. A potential limitation is that our ILM access variable (constructed with ownership data) is static because Amadeus database only reports the ultimate owner data for the last available year. However, we do believe that ownership patterns remain stable over time, as our sample is mostly made up of private firms and small business groups. Here, exploring how different labour market frictions affect the dynamics of group affiliation opens interesting paths for future research.

Relying on SIC codes to capture group ILM access can also be seen as a shortcoming. For instance, we consider that a group firm has no ILM access if it operates in the same region as other three sister companies but does not share the same two-digit SIC code with any of them. However, two group firms which are classified as unrelated according to the two-digit SIC code classification might be in fact vertically related and they could actually redeploy workers who have knowledge and skills that are applicable throughout the value chain of a product/service. Nonetheless, this potential shortcoming, if anything, should bias our analyses against finding support for the proposed hypotheses to the extent that we might be including in the group of firms without ILM access companies that indeed benefit from this type of organizational market.

A last weakness of our work is that, as much of the business group literature, firm’s affiliation to a group (which subsequently affects our ILM access measure) is identified on the basis of ownership and control relationships. However, even without ownership ties, some firms can be associated by multiple links, such as strategic alliances, franchising, subcontracting, and/or social relations (like family ties) through which they can coordinate to share resources and achieve mutual objectives (Granovetter, 1995; Lincoln et al., 2017). Studying if the access to network-internal resources provided by such non-ownership ties substitute/complement external markets is a promising avenue for future studies. It would be similarly interesting to investigate the position of the affiliate within the group, especially in the case of pyramidal structures (Belenzon et al., 2019b). Considering the affiliate’s position could unpack different degrees to which affiliates participate in, identify with, and are controlled by the group—which in turn reflects the extent to which they have more/less access to group-wide resources.

Finally, another promising avenue for future research would be to analyse which characteristics of ILMs generate a higher productivity premium. For instance, asymmetries in company size within the ILM network may affect intra-group reallocations. Workers may find it more appealing to be redeployed to a larger company in the ILM network but may resist being redeployed to a smaller company. Smaller companies are typically less attractive and secure employers than larger ones. Additionally, moving from a large to a smaller company may result in a loss of bargaining power, as large companies often have unions or employee associations negotiating on behalf of their members. As a result, in ILMs with asymmetries in company size, the redeployment of redundant employees may only be feasible in one direction (from small to large companies), whereas in ILMs between companies of similar size, it can occur between all companies in any direction, potentially increasing the productivity premium documented in this work.

### Practical implications

Our work has some practical implications for managers, policymakers, and trade union representatives. Being able to swiftly adapt the workforce to changing economic conditions is more important than ever in the post-COVID-19 scenario, where record employee attrition rates and labour shortages disrupt SMEs everywhere. In this context, many SMEs may leverage their group membership to generate value in terms of a superior ability to adjust and maximize the efficiency of human capital. Specifically, our findings help managers by emphasising the conditions under which one can expect to see ILMs of business groups as a labour-productivity advantage. We provide evidence that internally reallocating workers is more feasible and valuable in contexts associated with low employee bargaining power. Therefore, managers in business groups should consider bargaining power when committing resources to develop formal policies that support/foster employee intragroup redeployment. For example, promoting a corporate culture that views group internal reallocations positively is more likely to pay off in subnational regions with lower labour market tightness and/or in industries with lower job vacancy rates. Other policy examples can be (i) including special provisions where redundant employees of a group firm “A” have the opportunity to apply for a job in a sister company “B” before group-external candidates are considered; or (ii) providing support/training programs if the candidates to the redeployment do not meet all of the skills and experience requirements for a different position.

Our results are also relevant for trade union representatives and policymakers interested in enhancing job security and employee welfare. Both should be aware of the potential benefits and drawbacks of inter-firm ILMs for the reallocation of redundant employees. On the one hand, access to a business group’s ILM may provide higher job security and opportunities for employees to acquire new skills and gain from their involvement in different dimensions of the group’s operations. On the other hand, conducting efficiency-based inter-firm reallocations may lead to changes in employment conditions, such as reduced bargaining power and less favourable pay and benefits. Trade union representatives and policymakers can use this research to negotiate with employers within business groups regarding the creation of ILMs and the rules that govern internal reallocations. It is important for all parties involved to advocate for policies that provide SMEs with greater access to ILMs, which may lead to higher labour productivity and better employment opportunities, thus leading to a win-win situation for employee and employer. However, it is equally important to ensure that the rights and interests of workers are protected during intra-group reallocations.

## Supplementary Information

Below is the link to the electronic supplementary material.

Supplementary file1 (DOCX 72 KB)

## Data Availability

The data that support the findings of this study are available from Amadeus, a database developed by Bureau van Dijk (BvD), but restrictions apply to the availability of these data, which were used under licence for the current study and so are not publicly available. The data are, however, available from the authors upon reasonable request and with the permission of Bureau van Dijk (BvD).

## Footnotes

1 Official Journal L 018, January 21, 1997: p. 1–6. See https://eur-lex.europa.eu/legal-content/EN/TXT/?uri=CELEX%3A31996L0071&qid=1670940556957

2 More information on: https://ec.europa.eu/eurostat/web/structural-business-statistics/information-on-data/small-and-medium-sized-enterprises#:~:text=SMEs%20are%20defined%20as%20employing,more%20than%20EUR%2043%20million.

3 We restrict our sample only after having computed our *ILM access* measure as explained in Section 3.2.1.

4 In Table A8 of the Appendix, we show that our results are robust to including firms with fewer than 20 employees.

5 This threshold avoids the counterintuitive findings for situations involving two controlling firms when the threshold is lowered. Firms are classified as standalones if they have no ownership information.

6 Further details concerning the NUTS classification can be found on: https://ec.europa.eu/eurostat/web/nuts/background

7 The labour market slack rate is preferred over the unemployment rate, as it considers additional forms of “hidden unemployment” or “underemployment” that contribute to increase the frictions that prevent workers from putting their human capital on the market and that, according to our line or reasoning, affect the value of ILM access. It is worth noting that regional-level labour market slack allows for a more fine-grained understanding of the labour market conditions in which a firm and its staff are embedded than national slack figures do. Our European context offers a particularly apposite research setting, where there exits rich labour market conditions variation (both between countries and among subnational regions in those countries). For more details, visit: https://www.eurofound.europa.eu/observatories/eurwork/industrial-relations-dictionary/labour-market-slack

8 Further information available on: https://ec.europa.eu/eurostat/web/structural-business-statistics

9 More information on: https://cohesiondata.ec.europa.eu/stories/s/Regional-Competitiveness-Index-2019/363v-4uq6/

10 Specifically, ILM access is time invariant and some of our measures of labour bargaining power vary substantially across regions and/or countries but little over time, i.e., there is little within-panel variation. This feature is recurrent in prior works on how institutions affect the performance of group affiliated firms (see, e.g., Khanna & Palepu, 2000; Manikandan & Ramachandran, 2015).

11 More information can be found on: https://ec.europa.eu/eurostat/statistics-explained/index.php?title=Glossary:Knowledge_Intensive_Activity_(KIA)

12 We measure KIA group ties as the total number of group employees working for other sister companies that operate in a KIA. Similarly, we measure International group ties as the total number of group employees working for other sister companies that operate in a country other than the country of origin of the focal firm.

13 The seven largest countries in terms of area in the sample are France (551.7 K km^2^), Spain (506 K km^2^), Sweden (450.3 K km^2^), Germany (357.1 K km^2^), Finland (338.4 K km^2^), Poland (312.7 K km^2^), and Italy (301.3 K km^2^).
