# Supplementary material for: Business groups’ internal labour markets and SME labour productivity
Source: Small Bus Econ (Dordr). 2023 May 17:1–19. Online ahead of print. doi: 10.1007/s11187-023-00780-4 (PMC10189715; doi:10.1007/s11187-023-00780-4)
Supplement: Supplementary file 1 — Supplementary file1 (DOCX 72 KB) [file 11187_2023_780_MOESM1_ESM.docx]

**Appendix**

| **Table A1.** Correlation matrix of the main variables used in the regression analyses | | | | | | | | | | | | | | | | | |
| --- | --- | --- | --- | --- | --- | --- | --- | --- | --- | --- | --- | --- | --- | --- | --- | --- | --- |
|  | **1** | **2** | **3** | **4** | **5** | **6** | **7** | **8** | **9** | **10** | **11** | **12** | **13** | **14** | **15** | **16** |  |
| 1. Labour productivity | 1.00 |  |  |  |  |  |  |  |  |  |  |  |  |  |  |  |  |
| 2. ILM access | 0.10^***^ | 1.00 |  |  |  |  |  |  |  |  |  |  |  |  |  |  |  |
| 3. EPL index | 0.13^***^ | -0.05^***^ | 1.00 |  |  |  |  |  |  |  |  |  |  |  |  |  |  |
| 4. Collective bargaining coverage | 0.24^***^ | 0.03^***^ | 0.22^***^ | 1.00 |  |  |  |  |  |  |  |  |  |  |  |  |  |
| 5. Trade union density | 0.25^***^ | 0.03^***^ | 0.42^***^ | 0.55^***^ | 1.00 |  |  |  |  |  |  |  |  |  |  |  |  |
| 6. Job vacancy rate | 0.01^***^ | -0.02^***^ | 0.25^***^ | -0.29^***^ | 0.27^***^ | 1.00 |  |  |  |  |  |  |  |  |  |  |  |
| 7. Regional labour market tightness | 0.11^***^ | -0.03^***^ | 0.43^***^ | -0.24^***^ | 0.25^***^ | 0.45^***^ | 1.00 |  |  |  |  |  |  |  |  |  |  |
| 8. Firm size | -0.01^***^ | 0.07^***^ | 0.03^***^ | -0.14^***^ | -0.03^***^ | 0.14^***^ | 0.12^***^ | 1.00 |  |  |  |  |  |  |  |  |  |
| 9. Firm age | 0.21^***^ | -0.03^***^ | -0.01^***^ | 0.09^***^ | 0.06^***^ | -0.03^***^ | 0.04^***^ | 0.05^***^ | 1.00 |  |  |  |  |  |  |  |  |
| 10. Sales growth | -0.01^***^ | -0.00 | -0.01^***^ | 0.01^***^ | -0.01^***^ | 0.00^**^ | -0.03^***^ | -0.05^***^ | -0.23^***^ | 1.00 |  |  |  |  |  |  |  |
| 11. Capital intensity | 0.79^***^ | 0.06^***^ | 0.10^***^ | 0.27^***^ | 0.16^***^ | -0.06^***^ | 0.03^***^ | -0.03^***^ | 0.30^***^ | -0.09^***^ | 1.00 |  |  |  |  |  |  |
| 12. Leverage | 0.00^*^ | -0.06^***^ | 0.17^***^ | 0.22^***^ | 0.12^***^ | -0.06^***^ | -0.01^***^ | -0.03^***^ | -0.08^***^ | 0.02^***^ | -0.00 | 1.00 |  |  |  |  |  |
| 13. Num. group firms outside the region-industry | 0.17^***^ | 0.41^***^ | -0.01^***^ | 0.07^***^ | 0.09^***^ | 0.01^***^ | 0.02^***^ | 0.16^***^ | 0.01^***^ | -0.00 | 0.16^***^ | -0.11^***^ | 1.00 |  |  |  |  |
| 14. Industry labour productivity | 0.61^***^ | 0.01^***^ | 0.14^***^ | 0.23^***^ | 0.28^***^ | -0.01^***^ | 0.09^***^ | -0.00^**^ | 0.19^***^ | -0.04^***^ | 0.52^***^ | 0.00 | 0.11^***^ | 1.00 |  |  |  |
| 15. Regional GDP | 0.14^***^ | 0.05^***^ | 0.06^***^ | 0.43^***^ | -0.02^***^ | -0.30^***^ | -0.06^***^ | -0.04^***^ | 0.04^***^ | 0.00^**^ | 0.16^***^ | 0.11^***^ | 0.06^***^ | 0.06^***^ | 1.00 |  |  |
| 16. Regional competitiveness index | 0.26^***^ | 0.08^***^ | 0.16^***^ | 0.01^***^ | 0.46^***^ | 0.37^***^ | 0.57^***^ | 0.12^***^ | 0.09^***^ | -0.04^***^ | 0.14^***^ | -0.07^***^ | 0.14^***^ | 0.23^***^ | 0.15^***^ | 1.00 |  |
| + p < 0.10, * p < 0.05, ** p < 0.01, and *** p < 0.001 | | | | | | | | | | | | | | | | | |

| **Table A2.** Robustness check: ILM access defined in terms of region (NUTS 2) and three-digit SIC code | | | | | | | | |
| --- | --- | --- | --- | --- | --- | --- | --- | --- |
| **DV:** | **Labour productivity** | | | | | | | |
| **Employee bargaining power measure:** |  |  | **EPL strictness** | **Collective bargaining coverage** | **Trade union density** | **Job vacancy rate** | **Regional labour market tightness** |  |
| **Model:** | **(1)** | **(2)** | **(3)** | **(4)** | **(5)** | **(6)** | **(7)** |  |
| Firm size | 0.059^***^ | 0.059^***^ | 0.059^***^ | 0.066^***^ | 0.059^***^ | 0.061^***^ | 0.058^***^ |  |
|  | (0.003) | (0.003) | (0.003) | (0.003) | (0.003) | (0.003) | (0.003) |  |
| Firm age | 0.051^***^ | 0.051^***^ | 0.051^***^ | 0.047^***^ | 0.052^***^ | 0.051^***^ | 0.050^***^ |  |
|  | (0.002) | (0.002) | (0.002) | (0.002) | (0.002) | (0.002) | (0.002) |  |
| Sales growth | 0.215^***^ | 0.215^***^ | 0.213^***^ | 0.209^***^ | 0.208^***^ | 0.216^***^ | 0.215^***^ |  |
|  | (0.003) | (0.003) | (0.003) | (0.003) | (0.003) | (0.003) | (0.003) |  |
| Capital intensity | 0.372^***^ | 0.372^***^ | 0.374^***^ | 0.387^***^ | 0.381^***^ | 0.373^***^ | 0.372^***^ |  |
|  | (0.002) | (0.002) | (0.002) | (0.002) | (0.002) | (0.002) | (0.002) |  |
| Num. group firms outside the region-industry | 0.008^***^ | 0.007^***^ | 0.007^***^ | 0.007^***^ | 0.008^***^ | 0.007^***^ | 0.007^***^ |  |
|  | (0.000) | (0.000) | (0.000) | (0.000) | (0.000) | (0.000) | (0.000) |  |
| Leverage | -0.005^***^ | -0.005^***^ | -0.005^***^ | -0.006^***^ | -0.005^***^ | -0.006^***^ | -0.005^***^ |  |
|  | (0.000) | (0.000) | (0.000) | (0.001) | (0.001) | (0.000) | (0.000) |  |
| Industry labour productivity | 0.243^***^ | 0.243^***^ | 0.241^***^ | 0.248^***^ | 0.231^***^ | 0.244^***^ | 0.244^***^ |  |
|  | (0.005) | (0.005) | (0.006) | (0.006) | (0.006) | (0.006) | (0.005) |  |
| Regional GDP | 0.014^***^ | 0.014^***^ | 0.014^***^ | 0.012^***^ | 0.022^***^ | 0.020^***^ | 0.011^***^ |  |
|  | (0.002) | (0.002) | (0.002) | (0.002) | (0.003) | (0.002) | (0.002) |  |
| Regional competitiveness index | 0.131^***^ | 0.131^***^ | 0.131^***^ | 0.146^***^ | 0.135^***^ | 0.100^***^ | 0.092^***^ |  |
|  | (0.005) | (0.005) | (0.005) | (0.005) | (0.005) | (0.005) | (0.006) |  |
| **ILM access** |  | **0.005^***^** | **0.005^***^** | **0.006^***^** | **0.007^***^** | **0.005^***^** | **0.006^***^** |  |
|  |  | **(0.001)** | **(0.001)** | **(0.001)** | **(0.001)** | **(0.001)** | **(0.001)** |  |
| Bargaining power |  |  | 0.030^***^ |  |  | 0.018^***^ | 0.679^***^ |  |
|  |  |  | (0.003) |  |  | (0.001) | (0.034) |  |
| **ILM access × Bargaining power** |  |  | **-0.005^***^** | **-0.013^***^** | **-0.022^***^** | **-0.002^***^** | **-0.072^***^** |  |
|  |  |  | **(0.002)** | **(0.002)** | **(0.006)** | **(0.000)** | **(0.007)** |  |
| Constant | 1.715^***^ | 1.718^***^ | 1.703^***^ | 1.782^***^ | 1.782^***^ | 1.666^***^ | 1.727^***^ |  |
|  | (0.255) | (0.255) | (0.254) | (0.242) | (0.259) | (0.257) | (0.257) |  |
| Observations | 639,675 | 639,675 | 625,919 | 532,269 | 527,996 | 609,962 | 639,548 |  |
| Firms | 119,801 | 119,801 | 119,313 | 109,694 | 101,853 | 113,150 | 119,776 |  |
| Wald chi square | 287519.983 | 288068.922 | 284941.700 | 263930.367 | 244319.654 | 277430.517 | 291641.990 |  |
| p-value | 0.000 | 0.000 | 0.000 | 0.000 | 0.000 | 0.000 | 0.000 |  |
| R^2^ | 0.719 | 0.720 | 0.718 | 0.724 | 0.713 | 0.722 | 0.720 |  |
| This table presents the results of rerunning the regressions displayed in Table 2 using an alternative variable for ILM access. We ran GLS random-effects panel models in which standard errors (in parentheses) are robust to heteroscedasticity and allow for serial correlation through clustering by firms. All regressions incorporate industry (two-digit SIC codes), country, and year dummies. Bold font highlights variables or interactions of interest. | | | | | | | |  |

| **Table A3.** Robustness check: ILM access defined in terms of NUTS1 and two-digit SIC code | | | | | | | | |
| --- | --- | --- | --- | --- | --- | --- | --- | --- |
| **DV:** | **Labour productivity** | | | | | | |  |
| **Employee bargaining power measure:** |  |  | **EPL strictness** | **Collective bargaining coverage** | **Trade union density** | **Job vacancy rate** | **Regional labour market tightness** |  |
| **Model:** | **(1)** | **(2)** | **(3)** | **(4)** | **(5)** | **(6)** | **(7)** |  |
| Firm size | 0.059^***^ | 0.059^***^ | 0.060^***^ | 0.066^***^ | 0.059^***^ | 0.061^***^ | 0.058^***^ |  |
|  | (0.003) | (0.003) | (0.003) | (0.003) | (0.003) | (0.003) | (0.003) |  |
| Firm age | 0.051^***^ | 0.051^***^ | 0.050^***^ | 0.047^***^ | 0.052^***^ | 0.051^***^ | 0.050^***^ |  |
|  | (0.002) | (0.002) | (0.002) | (0.002) | (0.002) | (0.002) | (0.002) |  |
| Sales growth | 0.215^***^ | 0.215^***^ | 0.213^***^ | 0.209^***^ | 0.208^***^ | 0.216^***^ | 0.215^***^ |  |
|  | (0.003) | (0.003) | (0.003) | (0.003) | (0.003) | (0.003) | (0.003) |  |
| Capital intensity | 0.372^***^ | 0.372^***^ | 0.375^***^ | 0.387^***^ | 0.381^***^ | 0.373^***^ | 0.372^***^ |  |
|  | (0.002) | (0.002) | (0.002) | (0.002) | (0.002) | (0.002) | (0.002) |  |
| Leverage | -0.005^***^ | -0.005^***^ | -0.005^***^ | -0.006^***^ | -0.005^***^ | -0.006^***^ | -0.005^***^ |  |
|  | (0.000) | (0.000) | (0.000) | (0.001) | (0.001) | (0.000) | (0.000) |  |
| Num. group firms outside the region-industry | 0.008^***^ | 0.006^***^ | 0.006^***^ | 0.006^***^ | 0.007^***^ | 0.006^***^ | 0.006^***^ |  |
|  | (0.000) | (0.000) | (0.000) | (0.000) | (0.000) | (0.000) | (0.000) |  |
| Industry labour productivity | 0.243^***^ | 0.243^***^ | 0.240^***^ | 0.248^***^ | 0.231^***^ | 0.244^***^ | 0.244^***^ |  |
|  | (0.005) | (0.005) | (0.006) | (0.006) | (0.006) | (0.006) | (0.005) |  |
| Regional GDP | 0.015^***^ | 0.014^***^ | 0.014^***^ | 0.012^***^ | 0.022^***^ | 0.020^***^ | 0.012^***^ |  |
|  | (0.002) | (0.002) | (0.002) | (0.002) | (0.003) | (0.002) | (0.002) |  |
| Regional competitiveness index | 0.132^***^ | 0.132^***^ | 0.131^***^ | 0.147^***^ | 0.136^***^ | 0.100^***^ | 0.093^***^ |  |
|  | (0.005) | (0.005) | (0.005) | (0.005) | (0.005) | (0.005) | (0.006) |  |
| **ILM access** |  | **0.009^***^** | **0.009^***^** | **0.010^***^** | **0.012^***^** | **0.008^***^** | **0.009^***^** |  |
|  |  | **(0.001)** | **(0.001)** | **(0.001)** | **(0.001)** | **(0.001)** | **(0.001)** |  |
| **Bargaining power** |  |  | **0.031^***^** |  |  | **0.018^***^** | **0.695^***^** |  |
|  |  |  | **(0.003)** |  |  | **(0.001)** | **(0.034)** |  |
| **ILM access × Bargaining power** |  |  | **-0.006^***^** | **-0.023^***^** | **-0.039^***^** | **-0.002^***^** | **-0.099^***^** |  |
|  |  |  | **(0.002)** | **(0.004)** | **(0.005)** | **(0.001)** | **(0.010)** |  |
| Constant | 1.712^***^ | 1.717^***^ | 1.701^***^ | 1.782^***^ | 1.786^***^ | 1.665^***^ | 1.725^***^ |  |
|  | (0.255) | (0.255) | (0.254) | (0.242) | (0.258) | (0.257) | (0.257) |  |
| Observations | 639,677 | 639,677 | 625,921 | 532,270 | 527,996 | 609,964 | 639,550 |  |
| Firms | 119,802 | 119,802 | 119,314 | 109,695 | 101,853 | 113,151 | 119,777 |  |
| Wald chi square | 287312.692 | 287766.862 | 284637.857 | 263476.990 | 244186.939 | 277212.775 | 291537.131 |  |
| p-value | 0.000 | 0.000 | 0.000 | 0.000 | 0.000 | 0.000 | 0.000 |  |
| R^2^ | 0.719 | 0.719 | 0.718 | 0.724 | 0.713 | 0.722 | 0.720 |  |
| This table presents the results of rerunning the regressions displayed in Table 2 using an alternative variable for ILM access. We ran GLS random-effects panel models in which standard errors (in parentheses) are robust to heteroscedasticity and allow for serial correlation through clustering by firms. All regressions incorporate industry (two-digit SIC codes), country, and year dummies. Bold font highlights variables or interactions of interest. | | | | | | | |  |

| **Table A4.** Robustness check: ILM access defined in terms of country and two-digit SIC code | | | | | | | | |
| --- | --- | --- | --- | --- | --- | --- | --- | --- |
| **DV:** | **Labour productivity** | | | | | | | |
| **Employee bargaining power measure:** |  |  | **EPL strictness** | **Collective bargaining coverage** | **Trade union density** | **Job vacancy rate** | **Regional labour market tightness** |  |
| **Model:** | **(1)** | **(2)** | **(3)** | **(4)** | **(5)** | **(6)** | **(7)** |  |
| Firm size | 0.060^***^ | 0.058^***^ | 0.059^***^ | 0.066^***^ | 0.059^***^ | 0.061^***^ | 0.058^***^ |  |
|  | (0.003) | (0.003) | (0.003) | (0.003) | (0.003) | (0.003) | (0.003) |  |
| Firm age | 0.050^***^ | 0.051^***^ | 0.051^***^ | 0.047^***^ | 0.052^***^ | 0.051^***^ | 0.050^***^ |  |
|  | (0.002) | (0.002) | (0.002) | (0.002) | (0.002) | (0.002) | (0.002) |  |
| Sales growth | 0.215^***^ | 0.215^***^ | 0.213^***^ | 0.209^***^ | 0.208^***^ | 0.216^***^ | 0.215^***^ |  |
|  | (0.003) | (0.003) | (0.003) | (0.003) | (0.003) | (0.003) | (0.003) |  |
| Capital intensity | 0.373^***^ | 0.372^***^ | 0.374^***^ | 0.387^***^ | 0.381^***^ | 0.373^***^ | 0.371^***^ |  |
|  | (0.002) | (0.002) | (0.002) | (0.002) | (0.002) | (0.002) | (0.002) |  |
| Num. group firms outside the country-industry | 0.007^***^ | 0.008^***^ | 0.008^***^ | 0.008^***^ | 0.009^***^ | 0.008^***^ | 0.008^***^ |  |
|  | (0.000) | (0.000) | (0.000) | (0.000) | (0.000) | (0.000) | (0.000) |  |
| Leverage | -0.005^***^ | -0.005^***^ | -0.005^***^ | -0.006^***^ | -0.005^***^ | -0.006^***^ | -0.005^***^ |  |
|  | (0.000) | (0.000) | (0.000) | (0.001) | (0.001) | (0.000) | (0.000) |  |
| Industry labour productivity | 0.243^***^ | 0.243^***^ | 0.241^***^ | 0.248^***^ | 0.231^***^ | 0.244^***^ | 0.244^***^ |  |
|  | (0.005) | (0.005) | (0.006) | (0.006) | (0.006) | (0.006) | (0.005) |  |
| Regional GDP | 0.015^***^ | 0.014^***^ | 0.014^***^ | 0.011^***^ | 0.022^***^ | 0.019^***^ | 0.011^***^ |  |
|  | (0.002) | (0.002) | (0.002) | (0.002) | (0.003) | (0.002) | (0.002) |  |
| Regional competitiveness index | 0.133^***^ | 0.131^***^ | 0.130^***^ | 0.146^***^ | 0.135^***^ | 0.100^***^ | 0.091^***^ |  |
|  | (0.005) | (0.005) | (0.005) | (0.005) | (0.005) | (0.005) | (0.006) |  |
| **ILM access** |  | **0.006^***^** | **0.006^***^** | **0.006^***^** | **0.007^***^** | **0.006^***^** | **0.006^***^** |  |
|  |  | **(0.000)** | **(0.000)** | **(0.000)** | **(0.000)** | **(0.000)** | **(0.000)** |  |
| Bargaining power |  |  | 0.030^***^ |  |  | 0.018^***^ | 0.674^***^ |  |
|  |  |  | (0.003) |  |  | (0.001) | (0.034) |  |
| **ILM access × Bargaining power** |  |  | **-0.003^***^** | **-0.005^***^** | **-0.011^***^** | **-0.001^***^** | **-0.025^***^** |  |
|  |  |  | **(0.001)** | **(0.001)** | **(0.002)** | **(0.000)** | **(0.003)** |  |
| Constant | 1.700^***^ | 1.723^***^ | 1.708^***^ | 1.787^***^ | 1.785^***^ | 1.670^***^ | 1.731^***^ |  |
|  | (0.255) | (0.255) | (0.254) | (0.242) | (0.259) | (0.257) | (0.257) |  |
| Observations | 639,675 | 639,675 | 625,919 | 532,269 | 527,996 | 609,962 | 639,548 |  |
| Firms | 119,801 | 119,801 | 119,313 | 109,694 | 101,853 | 113,150 | 119,776 |  |
| Wald chi square | 286905.207 | 287911.616 | 284828.991 | 263646.111 | 244119.473 | 277311.930 | 291417.426 |  |
| p-value | 0.000 | 0.000 | 0.000 | 0.000 | 0.000 | 0.000 | 0.000 |  |
| R^2^ | 0.719 | 0.720 | 0.718 | 0.724 | 0.713 | 0.722 | 0.720 |  |
| This table presents the results of rerunning the regressions displayed in Table 2 using an alternative variable for ILM access. We ran GLS random-effects panel models in which standard errors (in parentheses) are robust to heteroscedasticity and allow for serial correlation through clustering by firms. All regressions incorporate industry (two-digit SIC codes), country, and year dummies. Bold font highlights variables or interactions of interest. | | | | | | | | |

| **Table A5.** Robustness check: ILM access defined in terms of country and two-digit SIC code, and excluding countries with the largest geographical areas | | | | | | |
| --- | --- | --- | --- | --- | --- | --- |
| **DV:** | **Labour productivity** | | | | | |
| **Countries excluded:** | **France, Spain, and Sweden** | | **France, Spain, Sweden, Germany, and Finland** | | **France, Spain, Sweden, Germany, Finland, Poland, and Italy** | |
| **Model:** | **(1)** | **(2)** | **(3)** | **(4)** | **(5)** | **(6)** |
| Firm size | 0.058^***^ | 0.058^***^ | 0.066^***^ | 0.066^***^ | 0.057^***^ | 0.057^***^ |
|  | (0.003) | (0.003) | (0.003) | (0.003) | (0.004) | (0.004) |
| Firm age | 0.053^***^ | 0.053^***^ | 0.055^***^ | 0.055^***^ | 0.014^**^ | 0.014^**^ |
|  | (0.002) | (0.002) | (0.003) | (0.003) | (0.005) | (0.005) |
| Sales growth | 0.214^***^ | 0.214^***^ | 0.216^***^ | 0.216^***^ | 0.291^***^ | 0.291^***^ |
|  | (0.003) | (0.003) | (0.003) | (0.003) | (0.005) | (0.005) |
| Capital intensity | 0.389^***^ | 0.389^***^ | 0.391^***^ | 0.391^***^ | 0.350^***^ | 0.350^***^ |
|  | (0.003) | (0.003) | (0.003) | (0.003) | (0.004) | (0.004) |
| Leverage | -0.006^***^ | -0.006^***^ | -0.007^***^ | -0.007^***^ | -0.007^***^ | -0.007^***^ |
|  | (0.001) | (0.001) | (0.001) | (0.001) | (0.001) | (0.001) |
| Num. group firms outside the country-industry | 0.009^***^ | 0.008^***^ | 0.010^***^ | 0.009^***^ | 0.008^***^ | 0.007^***^ |
|  | (0.000) | (0.000) | (0.000) | (0.000) | (0.001) | (0.001) |
| Industry labour productivity | 0.230^***^ | 0.231^***^ | 0.236^***^ | 0.236^***^ | 0.238^***^ | 0.238^***^ |
|  | (0.006) | (0.006) | (0.007) | (0.007) | (0.008) | (0.008) |
| Regional GDP | 0.019^***^ | 0.018^***^ | 0.015^***^ | 0.014^***^ | 0.004 | 0.003 |
|  | (0.003) | (0.003) | (0.003) | (0.003) | (0.007) | (0.007) |
| Regional competitiveness index | 0.181^***^ | 0.179^***^ | 0.193^***^ | 0.191^***^ | 0.187^***^ | 0.187^***^ |
|  | (0.007) | (0.007) | (0.007) | (0.007) | (0.009) | (0.009) |
| **ILM access** |  | **0.008^***^** |  | **0.008^***^** |  | **0.006^***^** |
|  |  | **(0.001)** |  | **(0.001)** |  | **(0.001)** |
| Constant | 1.628^***^ | 1.636^***^ | 1.517^***^ | 1.519^***^ | 2.744^***^ | 2.743^***^ |
|  | (0.327) | (0.327) | (0.329) | (0.328) | (0.335) | (0.335) |
| Observations | 431,878 | 431,878 | 400,994 | 400,994 | 194,047 | 194,047 |
| Firms | 80,874 | 80,874 | 73,923 | 73,923 | 34,044 | 34,044 |
| Wald chi square | 216375.216 | 216939.143 | 197189.653 | 197508.860 | 102079.574 | 102129.439 |
| p-value | 0.000 | 0.000 | 0.000 | 0.000 | 0.000 | 0.000 |
| R^2^ | 0.744 | 0.744 | 0.742 | 0.742 | 0.731 | 0.731 |
| This table provides some tests that can help minimize the distortions in our ILM access measure that may be generated by the fact that many firms affiliated with a group are organized around the border of two or more regions within the same country. To mitigate this potential distortion in the calculation of ILM access, we examine whether the productivity premium observed in Table 2 persists when employing the ILM access variable constructed at the country-two-digit SIC code level while excluding countries with the largest surface area from the estimation sample. The seven largest countries in terms of area in the sample are France (551.7K km^2^), Spain (506K km^2^), Sweden (450.3K km^2^), Germany (357.1K km^2^), Finland (338.4K km^2^), Poland (312.7K km^2^), and Italy (301.3K km^2^). We ran GLS random-effects panel models in which standard errors (in parentheses) are robust to heteroscedasticity and allow for serial correlation through clustering by firms. All regressions incorporate industry (two-digit SIC codes), country, and year dummies. Bold font highlights variables or interactions of interest. | | | | | | |

| **Table A6.** Robustness check: ILM access defined in terms of employees rather than sister firms | | | | | | | | |
| --- | --- | --- | --- | --- | --- | --- | --- | --- |
| **DV:** | **Labour productivity** | | | | | | | |
| **Employee bargaining power measure:** |  |  | **EPL strictness** | **Collective bargaining coverage** | **Trade union density** | **Job vacancy rate** | **Regional labour market tightness** |  |
| **Model:** | **(1)** | **(2)** | **(3)** | **(4)** | **(5)** | **(6)** | **(7)** |  |
| Firm size | 0.059^***^ | 0.058^***^ | 0.059^***^ | 0.066^***^ | 0.059^***^ | 0.061^***^ | 0.058^***^ |  |
|  | (0.003) | (0.003) | (0.003) | (0.003) | (0.003) | (0.003) | (0.003) |  |
| Firm age | 0.051^***^ | 0.051^***^ | 0.050^***^ | 0.047^***^ | 0.052^***^ | 0.051^***^ | 0.050^***^ |  |
|  | (0.002) | (0.002) | (0.002) | (0.002) | (0.002) | (0.002) | (0.002) |  |
| Sales growth | 0.215^***^ | 0.215^***^ | 0.213^***^ | 0.209^***^ | 0.208^***^ | 0.216^***^ | 0.215^***^ |  |
|  | (0.003) | (0.003) | (0.003) | (0.003) | (0.003) | (0.003) | (0.003) |  |
| Capital intensity | 0.372^***^ | 0.372^***^ | 0.374^***^ | 0.387^***^ | 0.381^***^ | 0.373^***^ | 0.372^***^ |  |
|  | (0.002) | (0.002) | (0.002) | (0.002) | (0.002) | (0.002) | (0.002) |  |
| Leverage | -0.005^***^ | -0.005^***^ | -0.005^***^ | -0.006^***^ | -0.005^***^ | -0.006^***^ | -0.005^***^ |  |
|  | (0.000) | (0.000) | (0.000) | (0.001) | (0.001) | (0.000) | (0.000) |  |
| Num. group firms outside the region-industry | 0.008^***^ | 0.007^***^ | 0.007^***^ | 0.007^***^ | 0.008^***^ | 0.007^***^ | 0.007^***^ |  |
|  | (0.000) | (0.000) | (0.000) | (0.000) | (0.000) | (0.000) | (0.000) |  |
| Industry labour productivity | 0.243^***^ | 0.242^***^ | 0.240^***^ | 0.247^***^ | 0.230^***^ | 0.243^***^ | 0.244^***^ |  |
|  | (0.005) | (0.005) | (0.006) | (0.006) | (0.006) | (0.006) | (0.005) |  |
| Regional GDP | 0.015^***^ | 0.014^***^ | 0.014^***^ | 0.011^***^ | 0.022^***^ | 0.020^***^ | 0.011^***^ |  |
|  | (0.002) | (0.002) | (0.002) | (0.002) | (0.003) | (0.002) | (0.002) |  |
| Regional competitiveness index | 0.132^***^ | 0.133^***^ | 0.132^***^ | 0.147^***^ | 0.136^***^ | 0.100^***^ | 0.093^***^ |  |
|  | (0.005) | (0.005) | (0.005) | (0.005) | (0.005) | (0.005) | (0.006) |  |
| **ILM access** |  | **0.010^***^** | **0.010^***^** | **0.014^***^** | **0.012^***^** | **0.010^***^** | **0.008^***^** |  |
|  |  | **(0.001)** | **(0.001)** | **(0.001)** | **(0.001)** | **(0.001)** | **(0.001)** |  |
| **Bargaining power** |  |  | **0.029^***^** |  |  | **0.018^***^** | **0.688^***^** |  |
|  |  |  | **(0.003)** |  |  | **(0.001)** | **(0.034)** |  |
| **ILM access × Bargaining power** |  |  | **-0.002** | **-0.035^***^** | **-0.035^***^** | **-0.005^***^** | **-0.093^***^** |  |
|  |  |  | **(0.002)** | **(0.007)** | **(0.006)** | **(0.001)** | **(0.015)** |  |
| Constant | 1.711^***^ | 1.728^***^ | 1.713^***^ | 1.799^***^ | 1.802^***^ | 1.677^***^ | 1.736^***^ |  |
|  | (0.255) | (0.255) | (0.254) | (0.242) | (0.258) | (0.257) | (0.257) |  |
| Observations | 639,677 | 639,677 | 625,921 | 532,270 | 527,996 | 609,964 | 639,550 |  |
| Firms | 119,802 | 119,802 | 119,314 | 109,695 | 101,853 | 113,151 | 119,777 |  |
| Wald chi square | 287346.717 | 287864.682 | 284687.401 | 263474.790 | 243726.601 | 277419.458 | 291244.861 |  |
| p-value | 0.000 | 0.000 | 0.000 | 0.000 | 0.000 | 0.000 | 0.000 |  |
| R^2^ | 0.719 | 0.720 | 0.718 | 0.724 | 0.713 | 0.722 | 0.720 |  |
| This table presents the results of rerunning the regressions displayed in Table 2 using an alternative variable for ILM access. We ran GLS random-effects panel models in which standard errors (in parentheses) are robust to heteroscedasticity and allow for serial correlation through clustering by firms. All regressions incorporate industry (two-digit SIC codes), country, and year dummies. Bold font highlights variables or interactions of interest. | | | | | | | | |

| **Table A7.** Coarsened exact matching models | | | | | | | |
| --- | --- | --- | --- | --- | --- | --- | --- |
| **DV:** | **Labour productivity** | | | | | | |
| **Employee bargaining power measure:** |  |  | **EPL strictness** | **Collective bargaining coverage** | **Trade union density** | **Job vacancy rate** | **Regional labour market tightness** |
| **Model:** | **(1)** | **(2)** | **(3)** | **(4)** | **(5)** | **(6)** | **(7)** |
| Firm size | 0.060^***^ | 0.060^***^ | 0.059^***^ | 0.062^***^ | 0.052^***^ | 0.060^***^ | 0.060^***^ |
|  | (0.005) | (0.005) | (0.005) | (0.006) | (0.006) | (0.005) | (0.005) |
| Firm age | 0.022^***^ | 0.023^***^ | 0.023^***^ | 0.019^***^ | 0.024^***^ | 0.021^***^ | 0.022^***^ |
|  | (0.004) | (0.004) | (0.004) | (0.004) | (0.004) | (0.004) | (0.004) |
| Sales growth | 0.175^***^ | 0.176^***^ | 0.175^***^ | 0.176^***^ | 0.174^***^ | 0.179^***^ | 0.176^***^ |
|  | (0.007) | (0.007) | (0.007) | (0.007) | (0.007) | (0.007) | (0.007) |
| Capital intensity | 0.406^***^ | 0.406^***^ | 0.406^***^ | 0.420^***^ | 0.410^***^ | 0.411^***^ | 0.405^***^ |
|  | (0.005) | (0.005) | (0.005) | (0.005) | (0.005) | (0.005) | (0.005) |
| Num. group firms outside the region-industry | 0.004^***^ | 0.003^***^ | 0.003^***^ | 0.003^***^ | 0.004^***^ | 0.003^***^ | 0.003^***^ |
|  | (0.000) | (0.000) | (0.000) | (0.000) | (0.000) | (0.000) | (0.000) |
| Leverage | -0.004^***^ | -0.004^***^ | -0.003^***^ | -0.005^***^ | -0.004^**^ | -0.004^***^ | -0.004^***^ |
|  | (0.001) | (0.001) | (0.001) | (0.001) | (0.001) | (0.001) | (0.001) |
| Industry labour productivity | 0.245^***^ | 0.245^***^ | 0.239^***^ | 0.241^***^ | 0.238^***^ | 0.257^***^ | 0.245^***^ |
|  | (0.012) | (0.012) | (0.012) | (0.013) | (0.013) | (0.013) | (0.012) |
| Regional GDP | 0.017^***^ | 0.016^***^ | 0.015^***^ | 0.020^***^ | 0.029^***^ | 0.013^**^ | 0.021^***^ |
|  | (0.004) | (0.004) | (0.004) | (0.004) | (0.005) | (0.004) | (0.004) |
| Regional competitiveness index | 0.097^***^ | 0.095^***^ | 0.099^***^ | 0.093^***^ | 0.099^***^ | 0.095^***^ | 0.026^*^ |
|  | (0.010) | (0.010) | (0.010) | (0.011) | (0.011) | (0.010) | (0.013) |
| **ILM access** |  | **0.011^***^** | **0.011^***^** | **0.012^***^** | **0.015^***^** | **0.011^***^** | **0.011^***^** |
|  |  | **(0.001)** | **(0.001)** | **(0.001)** | **(0.001)** | **(0.001)** | **(0.001)** |
| Bargaining power |  |  | 0.036^***^ |  |  | 0.007^*^ | 0.775^***^ |
|  |  |  | (0.010) |  |  | (0.004) | (0.063) |
| **ILM access × Bargaining power** |  |  | **-0.008^***^** | **-0.017^***^** | **-0.024^***^** | **-0.002^*^** | **-0.067^***^** |
|  |  |  | **(0.002)** | **(0.004)** | **(0.006)** | **(0.001)** | **(0.010)** |
| Constant | 1.395^***^ | 1.382^***^ | 1.416^***^ | 1.288^***^ | 1.200^***^ | 1.346^***^ | 1.322^***^ |
|  | (0.093) | (0.093) | (0.093) | (0.100) | (0.104) | (0.096) | (0.093) |
| Observations | 109,717 | 109,717 | 108,467 | 89,736 | 89,490 | 103,000 | 109,708 |
| Firms | 40,263 | 40,263 | 39,751 | 34,386 | 32,505 | 37,961 | 40,261 |
| Wald chi square | 85671.949 | 86102.121 | 84165.053 | 78975.023 | 67297.948 | 82813.755 | 87083.203 |
| p-value | 0.000 | 0.000 | 0.000 | 0.000 | 0.000 | 0.000 | 0.000 |
| R^2^ | 0.701 | 0.702 | 0.702 | 0.705 | 0.694 | 0.706 | 0.703 |
| This table presents the results of rerunning the regressions displayed in Table 2 on the matched sample. We ran GLS random-effects panel models in which standard errors (in parentheses) are robust to heteroscedasticity and allow for serial correlation through clustering by firms. All regressions incorporate industry (two-digit SIC codes), country, and year dummies. Bold font highlights variables or interactions of interest. | | | | | | | |

| **Table A8.** Robustness check: Main results including SMEs with fewer than 20 employees | | | | | | | |
| --- | --- | --- | --- | --- | --- | --- | --- |
| **DV:** | **Labour productivity** | | | | | | |
| **Employee bargaining power measure:** |  |  | **EPL strictness** | **Collective bargaining coverage** | **Trade union density** | **Job vacancy rate** | **Regional labour market tightness** |
| **Model:** | **(1)** | **(2)** | **(3)** | **(4)** | **(5)** | **(6)** | **(7)** |
| Firm size | 0.091^***^ | 0.090^***^ | 0.090^***^ | 0.100^***^ | 0.091^***^ | 0.091^***^ | 0.090^***^ |
|  | (0.001) | (0.001) | (0.001) | (0.002) | (0.002) | (0.001) | (0.001) |
| Firm age | 0.012^***^ | 0.012^***^ | 0.012^***^ | 0.007^***^ | 0.009^***^ | 0.011^***^ | 0.010^***^ |
|  | (0.001) | (0.001) | (0.001) | (0.001) | (0.001) | (0.001) | (0.001) |
| Sales growth | 0.152^***^ | 0.152^***^ | 0.150^***^ | 0.147^***^ | 0.148^***^ | 0.152^***^ | 0.152^***^ |
|  | (0.001) | (0.001) | (0.001) | (0.001) | (0.001) | (0.001) | (0.001) |
| Capital intensity | 0.360^***^ | 0.360^***^ | 0.361^***^ | 0.368^***^ | 0.368^***^ | 0.361^***^ | 0.360^***^ |
|  | (0.001) | (0.001) | (0.001) | (0.001) | (0.001) | (0.001) | (0.001) |
| Leverage | -0.007^***^ | -0.007^***^ | -0.007^***^ | -0.007^***^ | -0.007^***^ | -0.007^***^ | -0.007^***^ |
|  | (0.000) | (0.000) | (0.000) | (0.000) | (0.000) | (0.000) | (0.000) |
| Num. group firms outside the region-industry | 0.007^***^ | 0.006^***^ | 0.006^***^ | 0.005^***^ | 0.006^***^ | 0.006^***^ | 0.006^***^ |
|  | (0.000) | (0.000) | (0.000) | (0.000) | (0.000) | (0.000) | (0.000) |
| Industry labour productivity | 0.263^***^ | 0.263^***^ | 0.257^***^ | 0.283^***^ | 0.252^***^ | 0.272^***^ | 0.266^***^ |
|  | (0.004) | (0.004) | (0.004) | (0.005) | (0.005) | (0.004) | (0.004) |
| Regional GDP | 0.026^***^ | 0.026^***^ | 0.026^***^ | 0.020^***^ | 0.030^***^ | 0.027^***^ | 0.030^***^ |
|  | (0.001) | (0.001) | (0.001) | (0.002) | (0.002) | (0.002) | (0.002) |
| Regional competitiveness index | 0.107^***^ | 0.106^***^ | 0.105^***^ | 0.136^***^ | 0.109^***^ | 0.095^***^ | 0.025^***^ |
|  | (0.003) | (0.003) | (0.003) | (0.003) | (0.003) | (0.003) | (0.004) |
| **ILM access** |  | **0.011^***^** | **0.011^***^** | **0.013^***^** | **0.015^***^** | **0.011^***^** | **0.012^***^** |
|  |  | **(0.001)** | **(0.001)** | **(0.001)** | **(0.001)** | **(0.001)** | **(0.001)** |
| **Bargaining power** |  |  | **0.023^***^** |  |  | **0.011^***^** | **0.744^***^** |
|  |  |  | **(0.003)** |  |  | **(0.001)** | **(0.020)** |
| **ILM access × Bargaining power** |  |  | **-0.007^***^** | **-0.013^***^** | **-0.036^***^** | **-0.003^***^** | **-0.104^***^** |
|  |  |  | **(0.002)** | **(0.003)** | **(0.004)** | **(0.001)** | **(0.008)** |
| Constant | 1.532^***^ | 1.538^***^ | 1.550^***^ | 1.468^***^ | 1.575^***^ | 1.494^***^ | 1.469^***^ |
|  | (0.204) | (0.204) | (0.204) | (0.221) | (0.212) | (0.204) | (0.204) |
| Observations | 1,903,384 | 1903384 | 1865813 | 1627326 | 1620500 | 1817031 | 1903160 |
| Firms | 357,646 | 357,646 | 355,390 | 331,669 | 318,004 | 339,656 | 357,601 |
| Wald chi square | 520047.771 | 521076.616 | 512047.711 | 477059.618 | 444772.268 | 502907.708 | 527992.168 |
| p-value | 0.000 | 0.000 | 0.000 | 0.000 | 0.000 | 0.000 | 0.000 |
| R^2^ | 0.618 | 0.619 | 0.616 | 0.620 | 0.606 | 0.623 | 0.620 |
| This table presents the results of rerunning the regressions displayed in Table 2 on a sample that also incorporate information about SMEs with less than 20 employees. We ran GLS random-effects panel models in which standard errors (in parentheses) are robust to heteroscedasticity and allow for serial correlation through clustering by firms. All regressions incorporate industry (two-digit SIC codes), country, and year dummies. Bold font highlights variables or interactions of interest. | | | | | | | |
